# Therapies targeting triple-negative breast cancer: a perspective on anti-FGFR

**DOI:** 10.3389/fonc.2024.1415820

**Published:** 2025-03-11

**Authors:** Jinhao Chen, Qianru Wang, Hongyan Wu, Xiaofei Huang, Chunyu Cao

**Affiliations:** ^1^ Hubei Key Laboratory of Tumor Microenvironment and Immunotherapy, China Three Gorges University, Yichang, Hubei, China; ^2^ Affiliated Renhe Hospital of China Three Gorges University, Yichang, Hubei, China; ^3^ College of Basic Medical Sciences, China Three Gorges University, Yichang, China

**Keywords:** TNBC, FGF/FGFR pathway, tumor microenvironment, targeted therapies, FGFR inhibitor

## Abstract

Triple-negative breast cancer (TNBC) is one of the subtypes with the worst prognosis due to tumour heterogeneity and lack of appropriate treatment. This condition is a consequence of the distinctive tumour microenvironment (TME). The TME is associated with factors such as the promotion of proliferation, angiogenesis, inhibition of apoptosis, suppression of the immune system and drug resistance. Therefore, remodelling the TME is critical for the treatment of TNBC. A key role in the formation of the TME is played by the fibroblast growth factor/fibroblast growth factor receptor(FGF/FGFR) signalling pathway. Thus, the FGFRs may be a potential target for treating TNBC. Over-activated FGFRs promote growth, migration and drug resistance in TNBC by influencing the onset of TME events, tumour angiogenesis and immune rejection. A thorough comprehension of the FGF/FGFR signalling pathway’s mechanism of action in the development of TNBC could offer valuable insights for discovering new therapeutic strategies and drug targets. Inhibiting the FGF/FGFR axis could potentially hinder the growth of TNBC and its drug resistance by disrupting crucial biological processes in the TME, such as angiogenesis and immune evasion. This review evaluates the potential of inhibiting the FGF/FGFR axis as a strategy for treating TNBC. It explores the prospects for developing related therapeutic approaches. This study explores the research and application prospects of the FGF/FGFR axis in TNBC. The aim is to provide guidance for further therapeutic research and facilitate the development of innovative approaches targeting TNBC.

## 1 Introduction

Breast cancer is a prevalent malignant tumour among women, with the highest incidence rate and the second-highest mortality rate. The incidence of breast cancer has been gradually increasing since 2000 (1). Triple-negative breast cancer (TNBC) is a subtype of breast cancer that accounts for approximately 15-20% of cases. It is characterised by the absence of estrogen receptor (ER), progesterone receptor (PR) and human epidermal growth factor receptor 2 (HER2) expression. At present, a number of targeted therapies for TNBC are currently undergoing clinical trials, with polyadenosine diphosphate ribose polymerase (PARP), cyclin-dependent kinase 4 and 6 (CDK4/6), Ak strain transforming (AKT), and fibroblast growth factor receptor (FGFR) being identified as potential therapeutic targets (2). In recent years, immune checkpoint inhibitors, such as PD-1, have shown significant progress in clinically treating tumours. However, due to the large individual differences in the expression levels of PD-L1 ligands in tumour cells of TNBC patients and the presence of PD-L1 glycosylation modifications, the response rate to PD-1 or PD-L1 blockade therapy in TNBC patients in the clinic is only about 18.5% (3, 4). The FDA has now approved a number of strategies for combining immune checkpoints with chemotherapeutic agents for the treatment of TNBC. Cancer-associated fibroblasts (CAFs) play a critical role in the tumour microenvironment (TME) of TNBC by synthesising and secreting components of the tumour extracellular matrix (ECM). The ECM not only supports the growth and spread of tumour cells but also acts as a mechanical and chemical barrier, preventing the penetration of immune cells and chemotherapeutic agents. The stiffness and structural features of the ECM can alter the mechanical properties of the TME, affecting tumour cell behaviour and signalling (5). The ECM can act as a barrier, limiting immune cell infiltration and hindering the effectiveness of chemotherapeutic agents in attacking tumour cells, leading to the emergence of drug resistance (6, 7). Alterations in vascular endothelial cell (ECs) function in the TME can contribute to tumour cell invasion and metastasis. Two main mechanisms, the promotion of angiogenesis and the alteration of EC function, are involved in this process (8). As a result, TNBC is considered one of the most challenging subtypes of breast cancer to treat, with higher lethality and the worst prognosis compared to other subtypes.

The hyperactivation of fibroblast growth factors(FGFs) and FGFRs is significant in the development and advancement of various human tumours, including TNBC (9). FGFs is mainly sourced from CAFs in the ECM, and tumour cells can also produce autocrine FGFs (7, 10, 11). Additionally, persistent activation can result from abnormally high levels of FGFRs expression on the surface of tumour cells or mutations (9). The two aforementioned aspects result in the over-activation of FGF and its receptor-mediated signalling pathway, thereby promoting the proliferation, invasion, and metastasis of tumour cells (11–13).

Recent research has shown significant progress in TNBC targeted therapy against the FGF/FGFR pathway (7, 8, 14). This paper provides a review of the molecular mechanism of FGF/FGFR in promoting the occurrence and development of TNBC, as well as the development of FGFR inhibitors and their progress in TNBC therapy. This paper discusses the possible pathways of FGFRs in the TME that are involved in tumor immune escape. It also explores the prospects of anti-TNBC therapeutic research targeting the FGF/FGFR pathway.

## 2 FGF/FGFR family

FGF is composed of 22 glycoproteins, including FGF (1-10) and FGF (16-23), which are widely distributed throughout the body in various tissues and organs. Additionally, FGF (11-14) are intracellular and non-secretory, and their functions require further exploration (15). In addition, there are 18 secreted FGFs that act as FGFR ligands, while 15 FGFs function as classical ligands by forming complexes with heparan sulfate proteoglycans (HSPGs) through paracrine secretion and then binding to FGFR. HSPGs mainly serve to stabilise the FGF-FGFR binding and protect FGFs from degradation. FGF19 and its subfamily (FGF21 and FGF23) are distributed throughout the body as endocrine FGFs in the bloodstream. In contrast to the other 15 classical ligands, FGF19 and its subfamily require binding to klotho proteins to form a co-receptor before achieving high-affinity binding with FGFR. This is due to their lack of affinity for HSPG (16).

The FGFR family comprises four members, FGFR1-4, which are highly conserved tyrosine kinase transmembrane receptors (RTKs). Additionally, there is a receptor, FGFR5 (also known as FGFL1), which only has FGF-binding capacity and lacks an intracellular kinase structural domain (17). The extracellular region of FGFR comprises three sub-structural domains that are similar to immunoglobulins, namely IgI, IgII, and IgIII. Between IgI and IgII, there are eight consecutive acidic residues, which are commonly referred to as the acidic box. The structural domains IgII and IgIII are essential for ligand binding. The receptor’s amino-terminal portion, which includes Ig I and the acidic box, is autoinhibitory. Heterodimers with different ligand binding specificities can be produced by splicing the extracellular fragment of Ig III of FGFR (1-3). IgIIIb and IgIIIc are expressed specifically in epithelial cells and mesenchyme, respectively. The transmembrane receptor for FGFR comprises three components: a transmembrane structural domain composed of an alpha helix, a tyrosine kinase motif with the ability to phosphorylate, and an intracellular region with a carboxyl terminus (9).

## 3 FGF/FGFR pathway

**Figure 1:**
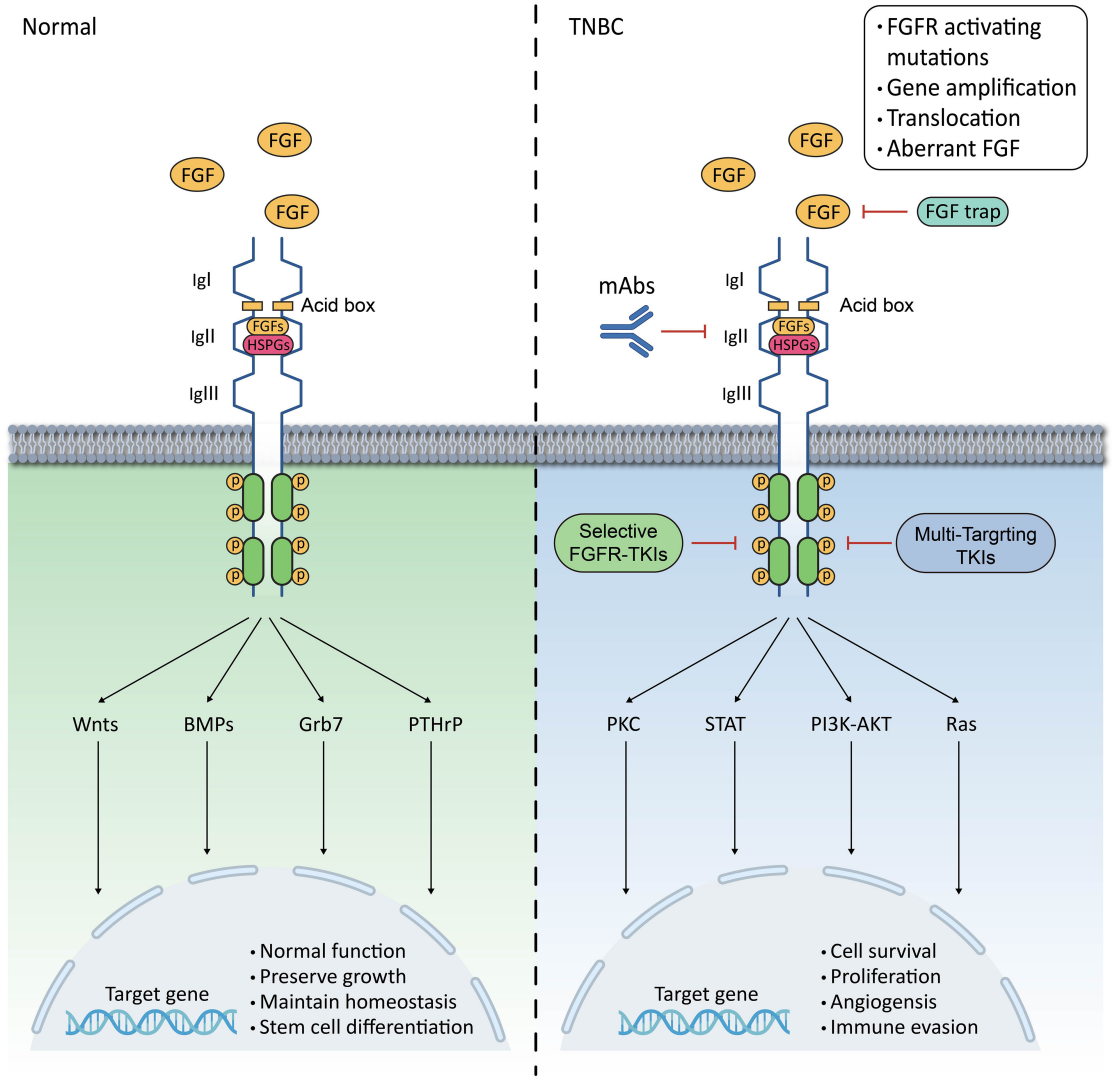
Schematic representation of FGF/FGFR signalling pathway in mammary epithelium and TNBC. The FGFR family has a basic structure comprising of three contiguous immunoprotein-like structural domains (Ig I, Ig II, and Ig III) in the extracellular and transmembrane regions, as well as intracellular membrane tyrosine kinase structural domains;In normal mammary epithelium, FGFR regulates Wnts, BMPs, Grb7, and PTHrP to maintain normal mammary epithelial function and sustain mammary epithelial stem cell differentiation;In TNBC, hyperactivation of the FGFR leads to overactivation of the PLCγ-PKC, STAT, PI3K-AKT, and Ras pathways, which trigger a range of biological behaviours in tumour cells;Hyperactivation of FGFR can result from activating mutations, gene amplification, chromosomal translocations, and aberrant ligands;Inhibition of FGFR can be achieved through TKIs, FGFR mAbs, and FGF traps, whether selective or non-selective.

FGFRs are a type of RTKs. When FGFs bind to inactive FGFR monomers, it leads to conformational changes in FGFRs. The intracellular tyrosine kinase structural domain of FGFRs is phosphorylated by phosphorylating their intracellular tyrosine residues, which triggers a conformational change in FGFRs, leading to dimerisation and activation of the receptor (18). The intracellular signalling cascade downstream of the signalling tyrosine kinases FGFRs is tightly regulated by specific linker proteins, such as FGFR substrate 2α (FRS2α), and regulators of the RAS-MAPK and PI3K-AKT pathways, such as the Sprouty (SPRY) protein. Additionally, it binds to phospholipase C-γ (PLC-γ) in an FRS2-independent manner and is involved in the STATs signalling pathway (18). Also the FGF/FGFR pathway is negatively feedback regulated by MAPK phosphatase 3 and SEF (expressed similarly to FGF) family members (19). Under normal physiological conditions, FGF/FGFR signalling plays a crucial role in maintaining homeostasis, growth, and development, as well as injury repair. FGFs act as broad-spectrum mitogens, involved in various cellular functions such as migration, proliferation, differentiation, and survival. Additionally, FGF/FGFR signalling is essential for embryonic development, metabolism, tissue homeostasis, and wound repair (20). The interaction between FGFs and FGFRs is complex. The binding of different FGFs to the receptor may produce opposite effects. Therefore, in some cases, the function of the FGF/FGFR signalling pathway is a combination of the functions of these molecules (20). In mammary epithelium, FGF/FGFR signalling regulates the activation of Wnts, BMPs, Grb7, PTHrP, and other factors. It is responsible for maintaining the differentiation of mammary stem cells and the normal function of mammary cells (21, 22) (**Figure 1**).

In pathological conditions, dysfunction of the FGF/FGFR signalling pathway can cause various organismal abnormalities, including genetic disorders such as congenital premature closure of the cranial sutures and dwarf syndrome, as well as neoplasms, chronic obstructive pulmonary disease, chronic kidney disease, obesity, and insulin resistance (20). This is closely related to the physiological activities of the FGF/FGFR signalling pathway in embryonic development and angiogenesis, where its over-activation or absence can lead to a range of pathological conditions. In cancer, dysregulation of the FGF/FGFR signalling pathway is frequently detected. Dysregulation of the FGFR cascade leads to the blockade of apoptosis and an increase in mitosis. This promotes epithelial-to-mesenchymal transition, leading to the proliferation, migration and infiltration of cancer cells. Among them, the over-activation of RAS-RAF-MAPK stimulates cell proliferation and differentiation, while the over-activation of PI3K-AKT inhibits apoptosis. Additionally, STATs are associated with the promotion of tumour invasion and metastasis, as well as the enhancement of tumour immune escape. Finally, the PLCγ signalling pathway is an important pathway in the regulation of tumour cell metastasis (9). In some diseases, such as acute lung injury (ALI) or acute respiratory distress syndrome (ARDS), the down-regulation of FGFR1 worsens lung injury, inflammatory infiltrates, and vascular infiltration, which can lead to disease progression (23). Overactivated FGFRs in TNBC promote tumour cell proliferation, migration, angiogenesis and immune evasion (7, 8, 12) (**Figure 1**).

## 4 Abnormal alterations in FGFR and TNBC tumourigenesis

The development of TNBC depends not only on the characteristics of the tumour cells but also on the TME. The TME refers to the interaction of the tumour cells with the surrounding stroma and non-cellular components. Similarly, the TME can vary among patients with the same tumour. FGFRs play an important role in mediating the interaction of breast cancer cells with the TME, promoting epithelial-mesenchymal transition (EMT) of tumour cells, among other functions. Furthermore, aberrantly activated FGFRs enhance signalling and promote the growth and anti-apoptotic capacity of tumour cells. Therefore, investigating the role of the FGF/FGFR signalling pathway in TNBC progression and its interactions with the entire tumour microenvironment is a major challenge for TNBC therapy (**Figure 1**).

### 4.1 Aberrant ligand signalling

As a ligand for FGFRs, FGF2 is considered the most closely related member of the FGF family to TNBC. TNBC patients with breast cancer typically exhibit higher levels of FGF2 than non-TNBC patients. Elevated levels of FGF2 have also been detected in plasma samples from TNBC patients and patients with other tumours, indicating that FGF2 may be an important tumour-associated factor (9, 24). A study found that a possible circRAD18-miR-3164-FGF2 axis promoted elevated levels of FGF2 in TNBC. Highly expressed FGF2 induced tumour growth, migration, angiogenesis, and migration of CAFs through activation of the ERK1/2-AKT-c-Rel pathway via its receptor FGFR1 (25). The FGF2/FGFR1 pathway promotes the transcription of the FGF-BP1 gene through the interaction of ϵ-sarcoglycan (SGCE) with Specificity Protein 1 (Sp1). This activation of the FGF2/FGFR1 signalling pathway promotes self-renewal and pluripotency of breast cancer stem cells (26).

### 4.2 FGFR genetic and epigenetic alterations in TNBC

FGFRs have been shown to be a potential oncogene. During carcinogenesis, FGFR signalling is triggered to be enhanced by genetic alterations, which include receptor amplification, mutations and chromosomal translocations (9). These alterations are prevalent in tumours and co-exist with other abnormal alterations. An analysis of large-scale next-generation sequencing of 4,853 tumours showed that FGFR abnormalities were present in approximately 7.1% of tumours, with FGFR amplification accounting for up to 66%, while point mutations and chromosomal heterozygosity accounted for 26% and 8%, respectively (27).

#### 4.2.1 Receptor amplification

FGFR family receptor amplification has been found in approximately 10-15% of TNBC cases (28). It has been found that approximately 5% of invasive TNBC cases have amplification of the FGFR1 locus (8q12) and increased levels of FGFR(1-4) mRNA expression, which is associated not only with an increase in gene copy number but also with resistance to and response to FGFR inhibitors (29). Furthermore, a negative correlation was found between survival and high FGFR expression in TNBC patients. High FGFR expression was also observed in other types of TNBC when compared to immune-excluded TNBC, which further highlights the significance of FGFR in immune rejection in TNBC (7). There is a correlation between FGFR1 signalling and endocrine resistance to treatment. A study discovered that FGFR1/2 amplification or activating mutations were present in around 40% of circulating tumour DNA (ctDNA) after treatment with CDK4/6 inhibitors. This correlates with the co-occurrence of FGFR1 amplification and altered PIK3CA gene activity in breast cancer (30). Furthermore, it was found that TNBC’s metastatic capacity was strongly associated with FGFR1 amplification. This is due to β3 integrin physically disrupting the interaction between FGFR1 and E-calmodulin, resulting in a significant increase in the redistribution of FGFR1 subcellular localization. This, in turn, enhances FGF2 signaling and strengthens the metastatic capacity (12). In addition, other families of amplification events, such as FGFR2 amplification and activation of other FGFR mutations, have been associated with the maintenance of tumour-initiating cells as well as high sensitivity to FGFR inhibitors. These genes have also been shown to have oncogenic properties or serve as potential therapeutic targets (14, 31). Furthermore, higher levels of FGFR4 expression were linked to a worse prognosis in TNBC (32). In conclusion, the amplification of FGFR1 and FGFR2 is prevalent in TNBC and is strongly linked to patient resistance to therapeutic agents and survival. Additionally, FGFR1 expression is an independent negative prognostic factor in TNBC (14).

#### 4.2.2 Activating mutations

Activating mutations in FGFR are a relatively rare occurrence compared to amplification, but they are one of the oncogenic features of TNBC. These mutations may result in various abnormalities in FGFR signalling pathways, including (i) dimerisation of FGFR bound in an irreversible form, (ii) over-activation of the structural domains of the receptor kinase, and (iii) alterations in the binding affinity of FGFR to FGF (9). Activating mutations can occur in different functional and structural domains of FGFR, while for epidermal growth factor receptor (EGFR) and vascular endothelial growth factor receptor (VEGFR), they only occur in the kinase structural domains (33). Compared to other types, mutations in FGFR1 are relatively infrequent. The two most common activating mutations in FGFR1 are N546K and K656E, located in the kinase structural domains, which result in increased kinase activation and conversion *in vitro*. The majority of point mutations in FGFRs occur in FGFR2. Interestingly, these somatically activated FGFR2 mutations occur predominantly in the transmembrane (Y375C, C382Y/R) and extracellular structural domains (S252W, W290C, P253R), rather than in kinase structural domains (N549H/K, K659E). The most common FGFR3 activating mutations are R248C and S249C, which occur in the extracellular structural domain, and G370C and Y373C, which occur in the transmembrane structural domain (34). Mutations can affect tumour progression and drug resistance. For instance, a C-terminal truncated FGFR2 isoform is more oncogenic than other mutations and is not affected by general inhibitors. Additionally, drug sensitivity varies depending on the mutation due to the presence of a proline-rich motif in the distal C-terminal end that binds to growth factor receptor-binding protein 2 (Grb2) and weakens the structural domain of the kinase. The proline-rich motif binds to Grb2 and reduces the activity of the kinase structural domain (35). Overexpression or aberrant activation of FGFR4 in TNBC leads to resistance to treatment with albumin-bound paclitaxel plus gemcitabine (36). Different types of mutations produce varying changes, albeit relatively small compared to amplification. These mutations play a role in drug resistance, oncogenicity, and epigenetic aspects of TNBC. Precision therapy, assisted by specific types of activating mutations, is a new type of treatment for TNBC (28, 35).

#### 4.2.3 Gene fusions

Gene fusion is a well-known factor that can cause cancer by joining two different genes together through chromosomal inversion or translocation to form a hybrid gene. Although the incidence of this anomaly is low, the individual differences in tumours it causes cannot be ignored. A study found that the fusion of two neighbouring genes, FGFR3 and TACC3, leads to excessive mitochondrial motility, which provides energy for rapid cell growth and thus promotes carcinogenesis. Experiments have shown that targeted therapy of this oncogenic factor can effectively stop tumour growth (37). The discovery of TNBC cell lines with an aberrant FGFR3-TACC3 fusion, which promotes oncogenic effects, is rare but may be useful for precision therapy of TNBC (38). It has also been shown that the fusion chaperones AFF3, CASP7 and CCDC6 aberrantly activate FGFR2 in TNBC (39). In conclusion, FGFR fusion occurs less frequently in TNBC, but it still contributes to TNBC heterogeneity.

#### 4.2.4 Abnormal epigenetic regulation in TNBC

The epigenetic regulation of FGFR1 involves DNA methylation and miRNA regulation. Studies have shown that the methylation level of the FGFR1 promoter is low in various solid tumours, particularly in breast, head and neck, oesophageal, bladder and endometrial cancers. This suggests that FGFR1 overexpression in different tumour types may be due to specific hypomethylated promoter sites. Analysis of the TCGA database in TNBC samples revealed a correlation between the mRNA expression of FGFR1, FGFR2, and FGF2 and the hypomethylation status of tumour cells (40). In the investigation of miRNA correlations, it was found that Hsa-mir-16-1 exhibited a significant negative correlation with FGFR1 in a subgroup of adenocarcinoma and squamous cell carcinoma patients. Additionally, widespread FGFR1 mRNA expression was observed in other solid tumour types, such as breast, head and neck, oesophageal, bladder and endometrial cancers. In these cancer types, the expression of eight different miRNAs was negatively correlated with FGFR1 mRNA levels (41). A study has found that the mechanism of TNBC resistance to FGFR inhibitors may be epigenetically related. Chronic treatment of TNBC with FGFR inhibitors results in high enhancer activation in open regions of chromatin that contain a large number of YAP/TEAD DNA-binding motifs. During drug resistance, enhancers of several amino acid transport proteins, such as SLC1A5, SLC7A5 and SLC3A2, are activated and can bind to YAP transcription factors. Furthermore, the chromatin binding profile of the SWI/SNF complex and its core protein BRG1 highly overlapped with the YAP/TEAD binding site. However, inhibition of FGFR resulted in the dissociation of BRG1 from chromatin. Knockdown of BRG1 in TNBC cells greatly facilitated YAP-dependent enhancer activation, as well as transcription of YAP target genes. YAP-recruited enhancers evicted the SWI/SNF complex, subsequently leading to activation of the mTORC1 complex. The results indicate that the increased expression of amino acid transporter proteins during drug resistance may be linked to epigenetic alterations (28, 42).

### 4.3 The function of FGF/FGFR in TNBC TME

**Figure 2:**
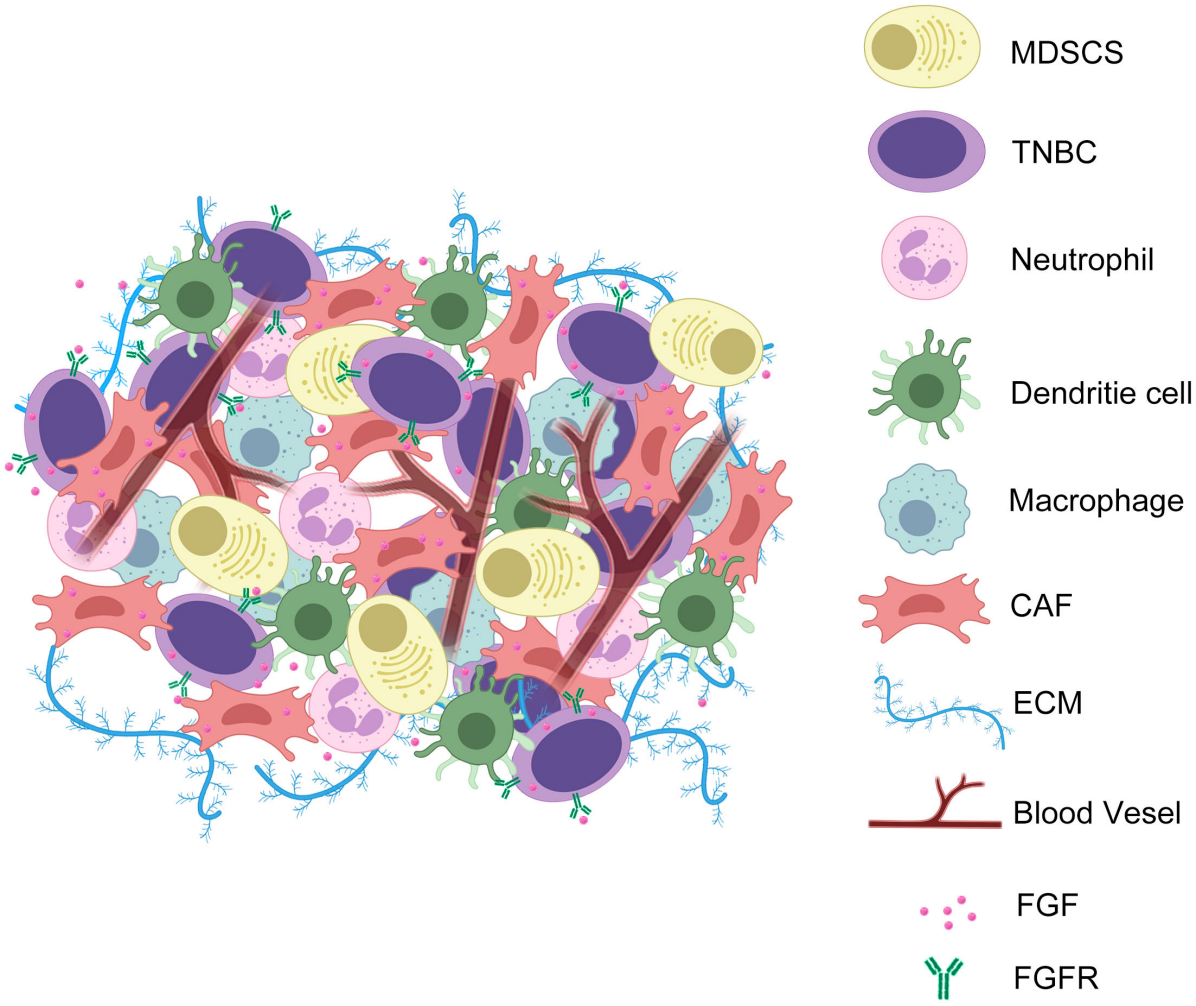
TNBC cells exchange FGF and FGFR signals with CAFs during EMT to promote angiogenesis, ECM secretion, and tumour immune evasion. In the TME of TNBC, the interaction of FGF with its ligands triggers a series of microenvironmental events. These include promoting the secretion of ECM by CAFs to form a barrier to the TME; promoting the infiltration of immune excluded-associated cells; promoting angiogenesis; promoting tumour cell proliferation and metastasis.

These interconnections are essential to the organism as a whole. Under physiological conditions, organisms rely on extensive cell-substrate interconnections to promote cell growth and development and maintain organismal homeostasis. However, in the TME, stable signalling molecules become dysregulated due to various factors in the cancer cells and tumour stroma. This dysregulation creates an environment that promotes tumourigenesis, growth, and migration. The FGF/FGFR system, an important branch of the growth factor family, is also affected by this dysregulation. Investigating the role of the FGF/FGFR signalling pathway in the TNBC TME and revealing the mechanisms by which it promotes tumour development has significant implications for the treatment of TNBC (**Figure 2**).

#### 4.3.1 Cancer-associated fibroblasts

Normal fibroblasts in the tumour stroma can be hijacked by cancer cells to form CAFs (6). CAFs play a crucial role in the TME of TNBC. Their main function is to synthesize and secrete the tumour ECM. The ECM provides support for tumour growth and spread, while also forming a mechanical and chemical barrier that prevents the penetration of immune cells and chemotherapeutic agents. CAFs promote tumour progression by secreting various extracellular matrix proteins, such as collagen fibres and fibronectin, which enhance cell proliferation, migration, and invasion. Meanwhile, the stiffness and structural characteristics of the ECM can alter the mechanical properties of the TME, thereby affecting the behaviour and signalling of tumour cells. CAFs play a crucial role in the TME of TNBC. Their main function is to synthesize and secrete the tumour ECM. The ECM provides support for tumour growth and spread, while also forming a mechanical and chemical barrier that prevents the penetration of immune cells and chemotherapeutic agents. CAFs promote tumour progression by secreting various extracellular matrix proteins, such as collagen fibres and fibronectin, which enhance cell proliferation, migration, and invasion. Meanwhile, the stiffness and structural characteristics of the ECM can alter the mechanical properties of the TME, thereby affecting the behaviour and signalling of tumour cells (6). In addition, chemokines in the ECM can also modulate the behaviour of tumour cells, for example by interacting with cell surface receptors to regulate cell proliferation, survival and metastasis. In addition to its direct effects on tumour cells, the ECM can form barriers that limit the infiltration of immune cells and prevent the effective penetration of chemotherapeutic agents into tumour cells, leading to the development of drug resistance (5, 7). CAFs are closely related to the FGF/FGFR system. Conventional FGF1 and unconventional FGF2 interact with FGFR3 and FGFR1 on tumours, respectively, which promotes the enhancement of tumour cell invasion mediated by CAFs (10). CAFs are activated forms of fibroblasts found in the tumour stroma. They secrete growth factors, such as TGF-β and FGF, in large quantities, which stimulate tumourigenesis, metastasis, and microenvironmental events, such as ECM remodelling and angiogenesis, in cancer cells (6). The level of FGF2 mRNA detected in CAFs was significantly higher than that in normal fibroblasts. FGF2 promoted the growth of CAFs through FGFR1 signalling, resulting in cancer cell proliferation, migration, invasion and angiogenesis (43). In TNBC, Hedgehog ligands produced by tumour cells reprogrammed CAFs. This reprogramming allowed tumour cells to acquire chemoresistance and a tumour stem cell phenotype through the expression of FGF5 and the production of fibrillar collagen (44). Recent studies have shown that TNBC exhibits significantly higher levels of CAFs-related protein expression compared to luminal types. Additionally, CAFs play a role in immune rejection of tumours. This is related to the fact that FGFR maintains the growth of CAFs and induces their secretion of vascular cell adhesion molecule 1 (VCAM-1) by promoting the MAPK/ERK signalling pathway. This, in turn, promotes the establishment of physical and chemical barriers by CAFs to prevent T-cell infiltration (7).

#### 4.3.2 Endothelial cells

Functional alterations of ECs in TME can contribute to the invasion and metastasis of tumour cells. This is achieved through two main mechanisms: promotion of angiogenesis and alteration of the function of vascular endothelial cells. ECs can participate in the process of neovascularisation, also known as angiogenesis or neovascularisation. In TNBC, tumour cells release angiogenic factors such as VEGF and FGF to stimulate angiogenesis. ECs respond by proliferating, migrating, and forming new vascular structures to provide blood supply and nutrition to the tumour. This process helps tumour cells grow and invade surrounding tissues, while also providing a gateway for cell escape and metastasis (8). Functional alterations in ECs can affect the properties and behaviour of blood vessels, thereby promoting invasion and metastasis of tumour cells. It is important to note that vascular endothelial cells normally form tightly connected vessel walls that are protective. In TNBC, tumour cells and other components release a variety of signalling molecules, such as inflammatory factors and proteases, which can alter the function of vascular endothelial cells. This leads to an increase in permeability, weakening of tight junctions, and decreased resistance of the vascular endothelium. As a result, tumour cells can cross the vessel wall and invade surrounding tissues or enter the circulatory system for metastasis (8). The FGF/FGFR and VEGF/VEGFR signalling pathways combine to promote angiogenesis (8, 45, 46). ECs receive signalling stimulation from FGF through the expression of FGFR and promote neoangiogenesis. Studies have reported that aberrant activation of the FGF/FGFR system enhances resistance to anti-VEGF therapy (47). Furthermore, FGFR regulates the secretion of VEGF in a MAPK-dependent manner. Subsequently, VEGF upregulates the expression of FGF. FGF induces the expression of VEGFR2 through an ERK1/2-dependent pathway. Without this interaction, the expression of VEGFR2 decreases rapidly (45). The study also showed that combined anti-FGFR and EGFR therapy suppressed tumour growth more effectively and enhanced the efficacy of anti-immunotherapy for tumours (46).

#### 4.3.3 Tumour-infiltrating immune cells

Tumour-infiltrating immune cells include a variety of cells such as lymphocytes, macrophages and myeloid-derived suppressor cells (MDSCs). M2-type tumor-associated macrophages (M2 TAMs) and MDSCs are an important part of the tumour’s ability to evade immune surveillance and destruction (48). In TNBC, the ligand protein PD-L1 is overexpressed on tumour cells and binds to PD-1 on T lymphocytes, leading to suppression of the immune recognition function of T lymphocytes (4, 49). This phenomenon leads to T-cell dysfunction or exhaustion in the TME, which reduces the immune attack on the tumour. However, with only 18.5% of TNBC patients responding to anti-PD-1/PD-L1 therapy, this type of immune cell-suppressing TME is a major challenge for TNBC treatment (3). Meanwhile,aberrantly activated FGFR signalling correlates with a non-T-cell inflammatory phenotype in tumours. FGFR expression in TNBC was negatively correlated with CD8+ T cells and M2-type tumor-associated macrophages(M1 TAMs)and positively correlated with fibroblasts and M2 TAMs (7). The study demonstrated that VEGFR and FGFR signalling suppressed the secretion of IFN-γ and granzyme B (GZMB) by T cells. Additionally, it significantly increased the expression of PD-1 in T cells and PD-L1 in tumour cells (46). Furthermore, experiments showed that bFGF and VEGFA signalling upregulated T-cell expression of PD-1, CTLA-4 and TIM-3, leading to T-cell depletion (50). At the same time, mice with defective FGF2 transmission were shown to have increased CD4+ and CD8+ T cell expression (51). The mechanisms of T-cell depletion have been extensively investigated, highlighting the role of the IFN-γ and peroxisome proliferator-activated receptor γ (PPARG) pathways and nuclear factor-κB (NF-κB) signalling. The FGF/FGFR pathway may lead to tumour immune rejection through these pathways (46). Furthermore, activation of FGFR1 triggers the expression of the chemokine CX3CL1 in the tumour microenvironment through NF-κB signalling. This, in turn, promotes the recruitment of macrophages, CD8+ T-cells, NK-cells, and dendritic cells (DCs) (52, 53). This evidence suggests that FGFR plays a significant role in tumour immunity. The infiltration of MDSCs can be reduced after the use of FGFR inhibitors in breast cancer treatment including TNBC. However, the mechanism by which FGFR mediates the action of MDSCs still needs to be further explored. It has been demonstrated that the use of FGFR inhibitors reduces proliferation and lung metastasis in TNBC and decreases the infiltration of MDSCs (54, 55). Related experiments have shown that FGFR reduces the mobilisation of MDSCs by decreasing the levels of granulocyte colony-stimulating factor (G-CSF) through mTOR signalling (56).

In conclusion, the FGF/FGFR signalling pathway is linked to the development of tumour cells and the formation of the tumour microenvironment. Further exploration of the mechanisms, cells, and signalling molecules involved in this process is necessary to provide a better theoretical basis for the treatment of TNBC.

## 5 FGFR-targeted therapeutic strategy for TNBC

FGFR1 expression is an independent prognostic marker for overall survival in TNBC patients. Trials have begun to use FGFR inhibitors in the therapeutic exploration of TNBC. The majority of TNBC are classified as immune-excluded tumours (57). Following the application of FGFR inhibitors, there is a significant increase in T-lymphocyte infiltration, which can convert immune-excluded TNBC into immune-inflammatory types (7). TNBC patients may benefit more from immunotherapeutic therapies, as research suggests. Additionally, inhibiting FGFR signalling, which is Overactivated in tumours, may inhibit tumour angiogenesis and suppress tumour growth (39).

### 5.1 The strategies for inhibiting FGF/FGFR

A variety of targeted FGFR inhibitors have been developed and some of them have entered clinical trials. Currently, the FDA has approved a variety of FGFR inhibitors on the market for the treatment of solid tumours (58, 59). Although FGFR-targeted therapies have not yet been approved for the treatment of breast cancer, many trials have begun to use FGFR inhibitors for the treatment of breast cancer, including TNBC (7, 42, 60, 61). FGFR inhibitors can be broadly classified into three categories: (i) small-molecule TKIs that selectively target the kinase structure of the FGFRs; (ii) small-molecule, multi-targeted tyrosine kinase inhibitor TKIs that block tyrosine kinase activity; and (iii) monoclonal antibody mAbs that block FGFRs as well as carry their ligands.

#### 5.1.1 Selective FGFR-TKIs

Selective FGFR-TKIs include single targeting of FGFR1-4 receptors or multiple receptors, but are restricted to the FGFR family. This is because although FGFR(1-4) are encoded by different genes, these four members are highly homologous, with sequence identity ranging from 56% to 71% (62). All of these receptors are expressed on the cell membrane surface and are stimulated and activated by extracellular signals. This high degree of similarity provides a good basis for small molecule targeted inhibitors. The basis of action for most selective FGFR-TKIs is competitive binding to the adenosine triphosphate (ATP) pocket of FGFRs. This structure is highly conserved among all kinase families, allowing most inhibitors to simultaneously inhibit multiple receptors (39). A study of TNBC patient-derived xenografts (PDX) and patient-derived organoids (PDO) treated with the selective tyrosine kinase inhibitors AZD4547 (FGFR1-3) and BLU9931 (FGFR4) showed favourable effects in the model of FGFRs expression enhancement, both in long-term and short-term use (14). Meanwhile, in TNBC tumour Vasculogenic mimicry (VM), AZD4547 disrupted the interconnection between ECs and TNBC cells, preventing the formation of a pro-angiogenic microenvironment (8). Futibatinib (TAS-120) has been approved for marketing by the FDA as an irreversible FGFR1-4 inhibitor and has demonstrated potent antiproliferative activity in a variety of cancer cell lines. These cell lines have FGFR genomic aberrations and exhibit anti-tumour activity in some non-FGFR-regulated PDX models (63). The TAS-120 breast cancer-related phase I clinical trial (NCT02052778) demonstrated significant anti-tumour activity, thereby establishing a foundation for subsequent breast cancer-related clinical trials (64). Infigratinib (BGJ398) is an FDA-approved and marketed FGFR1-4 inhibitor. Although it is similar to TAS-120 in terms of clinical application, it has not been used in TNBC. However, studies have shown that BGJ398 in combination with paclitaxel (PTX) significantly reduces resistance to certain chemotherapeutic agents in broad-spectrum ABCB1 overexpressing cancer cells, including TNBC cell lines (65). Similarly, Erdafitinib (JNJ42756493) is an FDA-approved inhibitor of FGFR1-4 for adult patients with locally advanced or metastatic uroepithelial carcinoma. Animal studies have shown that it also has inhibitory effects on TNBC cell lines (7). The Phase Ib clinical trial (NCT03238196) demonstrated the safety, tolerability, and anti-tumour activity of JNJ42756493 in combination with palbociclib for ER+/HER2-/FGFR-amplified metastatic breast cancer. Alofanib (RPT835) is a selective inhibitor of the variant against FGFR2. Experimental results indicate that RPT835 inhibits FGF-dependent tumour proliferation and reduces cell migration in FGFR2-expressing TNBC cell lines and PDX models (66).

#### 5.1.2 Multi-targeting TKIs

Multi-targeted tyrosine kinase inhibitors (TKIs) encompass a broad range of compounds that target the structural domains of FGFR, VEGFR, and platelet derived growth factor receptor (PDGFR) tyrosine kinases. These receptors are phylogenetically related and have a high degree of homology. *In vitro* experiments have shown that some non-selective VEGFR-targeting TKIs also inhibit FGFR (9). Tinengotinib (TT-00420) is a novel multikinase inhibitor that has been demonstrated to exert a significant inhibitory effect on Aurora A/B, FGFR1/2/3, VEGFRs, and JAK1/2 (67). The initial clinical trial of TT-00420 (NCT03654547) has indicated that it may have a favourable inhibitory effect on TNBC, which could serve as a potential targeted therapeutic agent for TNBC and has been shown to be consistent with the results of preclinical studies (67, 68). The results of a further Ib/II clinical trial, which includes patients with TNBC (NCT04742959), have yet to be published. However, it is anticipated that they will be promising. lucitanib (E3810) is a TKI targeting FGFR1-3, VEGFR1-3 and PDGFRα/β. Currently, the Phase II clinical trial of E3810 is the only preclinical trial in metastatic TNBC (NCT02202746), but the results are not yet available (69). However, another study found that the combination of E3810 and PTX resulted in significant tumour regression in a mouse model of advanced TNBC PDX (70). PD173074 is a potent inhibitor of FGFR1 and VEGFR2, showing inhibitory effects on TNBC in both *in vitro* and *in vivo* assays, as well as anti-tumour activity in FGFR-amplified TNBC cell lines (60, 61).

#### 5.1.3 mAbs and FGF trap

Although the development of tyrosine kinase activity inhibitors has been dominant, there has been an increasing focus on the research and development of monoclonal antibodies (mAbs) as therapeutic agents. mAbs are designed to hinder ligand interactions by inhibiting specific FGFRs or their dimerisation. GP369, a monoclonal antibody directed against the extracellular ligand-binding structural domain of FGFR2, showed promising results in FGFR2-IIIb subtype of breast cancer cell lines, demonstrating inhibition of tumour proliferation (71). Bemarituzumab (FPA144) is a humanised immunoglobulin G1 monoclonal antibody that specifically binds to the splice variant FGFR2b and inhibits the ligands FGF7, FGF10, and FGF22.It is also a monoclonal antibody in clinical trials (NCT02318329) and has demonstrated promising results in advanced solid tumours, as well as in FGFR2b overexpressed gastric adenocarcinoma and bladder cancer demonstrating promising applications and is safer than tyrosine kinase inhibitors (72).

FGF traps are a group of structurally heterogeneous molecules that act as FGFR decoys by binding FGFs in the extracellular environment. This prevents growth factor interaction with target cells. FGF traps are effective in avoiding the side effects produced by FGFR inhibitors, including hyperphosphatemia and retinal, nail, and skin toxicity. FP-1039, which consists of an extracellular domain of human FGFR1α-IIIc linked to the modified chain of human immunoglobulin G1 and the native Fc region, has demonstrated promising results in patients with advanced malignancies clinical trials(NCT00687505). Additionally, it has been shown to be safe and tolerable in breast cancer patients (73, 74). FGF traps are not currently used in TNBC, but they offer advantages over FGFR inhibitors that could be applied to therapeutic studies in TNBC.

**Figure 3:**
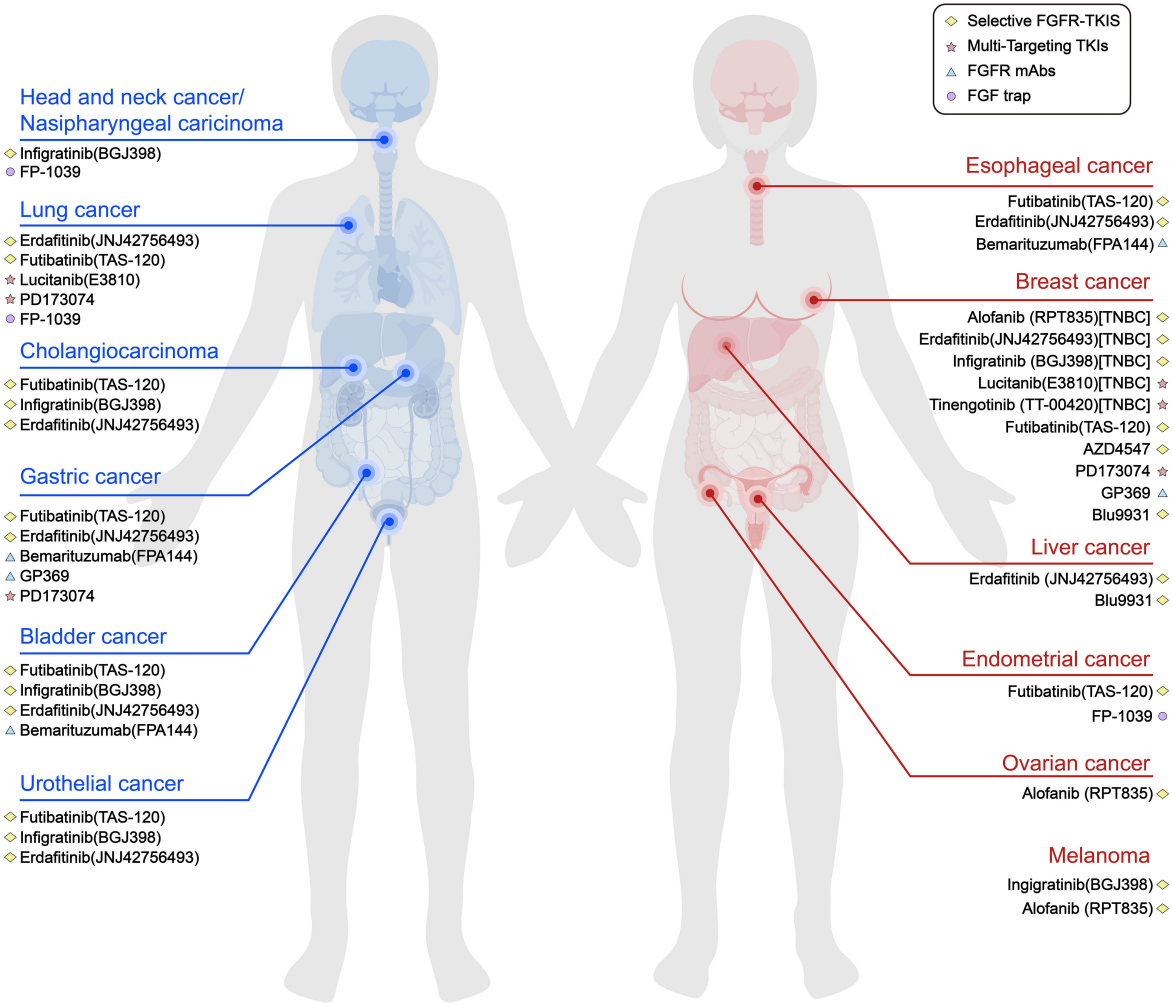
Current part of FGFR inhibitors in various types of solid tumours. Pathways that inhibit FGF/FGFR signalling include selective FGFR TKIs, multi-targeting TKIs, FGFR mAbs and FGF traps.To date, most FGFR inhibitors used in TNBC trials are TKIs.

FGFR inhibitors have shown value in TNBC as well as other tumours, both in clinical trials and in preclinical trials. The combination treatment approach can be either FGFR inhibitors alone or in combination with other therapies. In addition, there is a need to explore more rational pathways of FGF/FGFR signalling pathway inhibition and to continuously develop the potential of tyrosine kinase inhibitors and monoclonal antibodies for application. These efforts will provide better and reliable pathways for the treatment of TNBC (**Figure 3**).

### 5.2 The functional effects of FGF/FGFR inhibition in TNBC

T-cell rejection can facilitate tumour immune escape and resistance to immunotherapy. In order to inhibit the activation of the MAPK/ERK signalling pathway in CAFs, FGFR inhibitors are used to block FGFR. This inhibits the proliferation, migration and secretion of VCAM-1 in CAFs, disrupting the physical and chemical barriers established by CAFs and preventing T cell infiltration. This inhibits the proliferation, migration and secretion of VCAM-1 in CAFs, disrupting the physical and chemical barriers established by CAFs and preventing T cell infiltration. Furthermore, FGFR inhibitors have been shown to increase the infiltration of anti-tumour immune cells, such as CD8+ T cells and M1 TAMs, while inhibiting the infiltration of pro-tumour immune cells, such as MDSCs and M2 TAMs. Additionally, FGFR inhibitors enhance the anti-tumour activity of cytotoxic T lymphocytes (CTLs) in tumours, thereby improving the efficacy of immune checkpoint blockade (ICB) (7). In a combination therapy, Erdafitinib and anti-PD-1 oncology treatment demonstrated a synergistic anti-tumour effect. The synergistic effect was dependent on Erdafitinib-induced tumour cell killing, re-initiation, and enhanced anti-tumour T-cell responses through PD-1 blockade (75). FGFR has the potential to inhibit T-cell activation and infiltration, promote M2 TAMs transformation and recruitment, and maintain MDSCs through factors such as IFN-γ, GZMB, and chemokines. Over-activation of FGFR signalling inhibits the IFN-γ-stimulated JAK/STAT signalling pathway. Inhibition of FGFR releases FGFR-induced inhibition of JAK/STAT signalling and restores tumour cell responses to IFN-γ-activated tumours. It can be assumed that the over-activation of FGFR signalling is involved in the formation of tumour immune rejection (46). Furthermore, the data indicate that FGFR inhibitors could enhance the responsiveness of tumours to ICB therapy by influencing various stages of the ‘tumour-immune cycle’. This includes promoting the trafficking and infiltration of immune cells, activating T cells, reducing immune-suppressing cells, and enhancing tumour antigenicity (76).

Elevated levels of VEGFA and FGF can promote immune rejection of tumours. When stimulated, bFGF and VEGFA significantly increase the expression of PD-1, CTLA-4, and TIM-3 on T cells. They also inhibit the secretion of IFN-γ and GZMB in T cells and reduce T cell cytotoxicity. These effects on T cells were even more pronounced when bFGF and VEGFA were combined. The use of dual FGFR/VEGFR inhibitors can convert immunologically ‘cold’ tumours into ‘hot’ tumours, reducing tumour vascular density and restoring T cell function. This maintains tumour cell sensitivity to PD-1 monoclonal antibodies (8, 45, 50). FGFR1 and β3 integrins play important roles in EMT. Activation of Erk1/2 signalling is a necessary mechanism for disseminated breast cancer cells to overcome systemic dormancy and undergo metastatic growth. FGFR complexes with β3 integrins act as upstream mediators of Erk1/2 activation. EMT-mediated activation of FGFR1 and β3 integrin complexes promotes metastatic tumour growth by enhancing local focal adhesion kinase (FAK) activity. The experiment using the irreversible FGFR blocker FINN-4 significantly inhibited the growth of metastatic TNBC in a TNBC metastasis model (12). Approximately 30% of TNBC cases exhibit aberrant PI3K/mTOR signalling. The methylation of G protein-coupled receptor class C, member 5, A (GPRC5A) has been demonstrated to activate mTOR in TNBC, thereby promoting the development of liver metastases and enhancing resistance to chemotherapy drugs (77). However, inhibiting TORC1/2 leads to the acquisition of cancer stem cell (CSC) properties and resistance. Additionally, TORC1/2 inhibition upregulates FGFR1 expression, which activates Notch1 signalling through a TFAM-dependent mechanism. Activated Notch1 signalling is an important pathway for maintaining CSCs. Combination therapies that inhibit the FGFR-mitochondrial metabolism-Notch1 axis may then limit the growth of tumour stem cell subpopulations and enhance the anti-tumour effects of TORC1/2 inhibitors (78).

The effect of inhibiting the FGF/FGFR axis on various tumour therapies is increasingly being investigated in combination with other treatments. In many studies, such combination therapies have produced satisfactory results, but some combinations have failed to achieve the desired effect. Among many ICB therapies, most studies have used FGFR inhibitors in combination with anti-PD-1 drugs, and the mechanisms behind this need to be further investigated. Combinations with other drugs also need to be validated in future studies.

## 6 Discussion

Publicly available data shows that between 2016 and 2020, the number of solid tumours associated with FGFR worldwide increased from 4.4 million to 4.9 million, at a compound annual growth rate of 3.0%. By 2035, this number is expected to reach 6.8 million. This trend has driven research into the FGF/FGFR signalling pathway in tumourigenesis and development, and has also made the application of FGFR inhibitors in tumours a hot topic. FGF/FGFR is a valuable target in tumour therapy. In recent years, the FDA has expedited the approval of certain FGFR inhibitor-related drugs, which have demonstrated success in treating specific solid tumours. However, drugs targeting FGF/FGFR for TNBC therapy are still in the experimental stage.

The heterogeneity of TNBC tumours is a major challenge in their treatment. Targeted therapies remain challenging for TNBC due to molecular heterogeneity. However, each TNBC subtype is susceptible to specific therapies. The difficulty with FGFR inhibitors in TNBC is the need to characterise the heterogeneity of TNBC. However, FGFR is enriched in immune-excluded phenotypes of TNBC, making targeted therapy for different types of TNBC a viable option. In particular, the targeted use of FGFR inhibitors in different gene fusion types may significantly increase the number of cancer patients who may benefit from such therapy. Resistance to FGFR inhibitors is also an issue that should not be ignored, as TNBC may acquire resistance to FGFR inhibitors or other drugs through epigenetic and overexpression of FGFR, for example. Therefore, further research is needed to develop FGFR inhibitors suitable for TNBC or in combination with other drugs to circumvent the development of resistance. The variability in the mechanism of FGFR activation also suggests many issues that need to be improved, and many preclinical experiments show the importance of targeting individual differences. FGFR inhibitors alone or in combination with other agents have shown promising therapeutic effects in both clinical and preclinical studies.

In conclusion, TNBC is a highly challenging form of breast cancer when compared to other types. One potential avenue for therapy is to remodel the tumour microenvironment, transforming it from immune-excluded to immune-infiltrating, followed by adjuvant immunotherapy or other means. TNBC, as an immunosuppressive tumour, may benefit from additional immunotherapies and increased sensitivity to therapeutic means. This includes enhancing the anti-tumour effects of FGFR inhibitors themselves. However, a refined patient selection strategy is necessary to improve the efficacy of FGFR inhibitor-targeted therapy. Furthermore, it may be feasible to target common pathways of oncogene signalling as a strategy for treating TNBC. This can be achieved through comprehensive studies of the FGF/FGFR signalling pathway and TNBC oncogenic modalities.

Furthermore, the particular effects of distinct FGFR family members on TNBC are not fully investigated. The majority of clinical and preclinical studies are primarily focused on identifying the alterations in various FGFRs in TNBC and subsequently developing targeted therapies. It is necessary to investigate whether different FGFRs exhibit disparate effects on TNBC, as this could be crucial for the subsequent TNBC treatment study.

## References

[1] Siegel RL Giaquinto AN Jemal A. Cancer statistics, 2024. CA Cancer J Clin. (2024) 74:12–49. doi: 10.3322/caac.21820 38230766

[2] Ye F Dewanjee S Li Y Jha NK Zhe-Sheng C Kumar A. Advancements in clinical aspects of targeted therapy and immunotherapy in breast cancer. Mol Cancer. (2023) 22:105. doi: 10.1186/s12943-023-01805-y 37415164 PMC10324146

[3] Li CW Lim SO Chung EM Kim YS Park AH Yao J. Eradication of triple-negative breast cancer cells by targeting glycosylated PD-L1. Cancer Cell. (2018) 33:187–201. doi: 10.1016/j.ccell.2018.01.009 29438695 PMC5824730

[4] Zhang Y Chen H Mo H Hu X Gao R Zhao Y. Single-cell analyses reveal key immune cell subsets associated with response to PD-L1 blockade in triple-negative breast cancer. Cancer Cell. (2021) 39:1578–93. doi: 10.1016/j.ccell.2021.09.010 34653365

[5] Yang D Liu J Qian H Zhuang Q. Cancer-associated fibroblasts: from basic science to anticancer therapy. Exp Mol Med. (2023) 55:1322–32. doi: 10.1038/s12276-023-01013-0 PMC10394065 37394578

[6] Sahai E Astsaturov I Cukierman E DeNardo DG Egeblad M Evans RM. A framework for advancing our understanding of cancer-associated fibroblasts. Nat Rev Cancer. (2020) 20:174–86. doi: 10.1038/s41568-019-0238-1 PMC7046529 31980749

[7] Wu Y Yi Z Li J Wei Y Feng R Liu J. FGFR blockade boosts T cell infiltration into triple-negative breast cancer by regulating cancer-associated fibroblasts. Theranostics. (2022) 12:4564–80. doi: 10.7150/thno.68972 PMC9254240 35832090

[8] Morales-Guadarrama G Mendez-Perez EA Garcia-Quiroz J Avila E Ibarra-Sánchez MJ Esparza-López J. The inhibition of the FGFR/PI3K/Akt axis by AZD4547 disrupts the proangiogenic microenvironment and vasculogenic mimicry arising from the interplay between endothelial and triple-negative breast cancer cells. Int J Mol Sci. (2023) 24(18):13770. doi: 10.3390/ijms241813770 37762073 PMC10531243

[9] Babina IS Turner NC. Advances and challenges in targeting FGFR signalling in cancer. Nat Rev Cancer. (2017) 17:318–32. doi: 10.1038/nrc.2017.8 28303906

[10] Erdogan B Webb DJ. Cancer-associated fibroblasts modulate growth factor signaling and extracellular matrix remodeling to regulate tumor metastasis. Biochem Soc Trans. (2017) 45:229–36. doi: 10.1042/BST20160387 PMC5371349 28202677

[11] Ferguson HR Smith MP Francavilla C. Fibroblast growth factor receptors (FGFRs) and noncanonical partners in cancer signaling. Cells. (2021) 10(5):1201. doi: 10.3390/cells10051201 34068954 PMC8156822

[12] Brown WS Tan L Smith A Gray NS Wendt MK. Covalent targeting of fibroblast growth factor receptor inhibits metastatic breast cancer. Mol Cancer Ther. (2016) 15:2096–106. doi: 10.1158/1535-7163.MCT-16-0136 PMC5010989 27371729

[13] Wiedlocha A Haugsten EM Zakrzewska M. Roles of the FGF-FGFR signaling system in cancer development and inflammation. Cells. (2021) 10(9):2231. doi: 10.3390/cells10092231 34571880 PMC8471549

[14] Chew NJ Lim KST Nguyen EV Shin S-Y Yang J Hui MN. Evaluation of FGFR targeting in breast cancer through interrogation of patient-derived models. Breast Cancer Res. (2021) 23:82. doi: 10.1186/s13058-021-01461-4 34344433 PMC8336364

[15] Itoh N Ornitz DM. Fibroblast growth factors: from molecular evolution to roles in development, metabolism and disease. J Biochem. (2011) 149:121–30. doi: 10.1093/jb/mvq121 PMC3106964 20940169

[16] Chen L Fu L Sun J Huang Z Fang M Zinkle A. Structural basis for FGF hormone signalling. Nature. (2023) 618:862–70. doi: 10.1038/s41586-023-06155-9 PMC10284700 37286607

[17] Trueb B. Biology of FGFRL1, the fifth fibroblast growth factor receptor. Cell Mol Life Sci. (2011) 68:951–64. doi: 10.1007/s00018-010-0576-3 PMC11115071 21080029

[18] Ornitz DM Itoh N. The Fibroblast Growth Factor signaling pathway. Wiley Interdiscip Rev Dev Biol. (2015) 4:215–66. doi: 10.1002/wdev.2015.4.issue-3 PMC4393358 25772309

[19] Szybowska P Kostas M Wesche J Haugsten EM Wiedlocha A. Negative Regulation of FGFR (Fibroblast Growth Factor Receptor) Signaling. Cells. (2021) 10:1342. doi: 10.3390/cells10061342 34071546 PMC8226934

[20] Xie Y Su N Yang J Tan Q Huang S Jin M. FGF/FGFR signaling in health and disease. Signal Transduct Target Ther. (2020) 5:181. doi: 10.1038/s41392-020-00222-7 32879300 PMC7468161

[21] Lofgren KA Kenny PA. Grb7 knockout mice develop normally but litters born to knockout females fail to thrive. Dev Dyn. (2023) 253(7):677–89. doi: 10.1101/2023.06.29.546912 38140940

[22] Pond AC Bin X Batts T Roarty K Hilsenbeck S Rosen JM. Fibroblast growth factor receptor signaling is essential for normal mammary gland development and stem cell function. Stem Cells. (2013) 31:178–89. doi: 10.1002/stem.1266 PMC3690809 23097355

[23] Deng Y Huang X Hu Y Zhong W Zhang H Mo C. Deficiency of endothelial FGFR1 signaling via upregulation of ROCK2 activity aggravated ALI/ARDS. Front Immunol. (2023) 14:1041533. doi: 10.3389/fimmu.2023.1041533 36969192 PMC10036754

[24] Santolla MF Talia M Maggiolini M. S100A4 is involved in stimulatory effects elicited by the FGF2/FGFR1 signaling pathway in triple-negative breast cancer (TNBC) cells. Int J Mol Sci. (2021) 22(9):4720. doi: 10.3390/ijms22094720 33946884 PMC8124532

[25] Zou Y Zheng S Xiao W Xie X Yang A Gao G. circRAD18 sponges miR-208a/3164 to promote triple-negative breast cancer progression through regulating IGF1 and FGF2 expression. Carcinogenesis. (2019) 40:1469–79. doi: 10.1093/carcin/bgz071 31001629

[26] Qiu T Hou L Zhao L Wang X Zhou Z Yang C. SGCE promotes breast cancer stemness by promoting the transcription of FGF-BP1 by Sp1. J Biol Chem. (2023) 299:105351. doi: 10.1016/j.jbc.2023.105351 37838174 PMC10641673

[27] Helsten T Elkin S Arthur E Tomson BN Carter J Kurzrock R. The FGFR landscape in cancer: analysis of 4,853 tumors by next-generation sequencing. Clin Cancer Res. (2016) 22:259–67. doi: 10.1158/1078-0432.CCR-14-3212 26373574

[28] Orlando KA Wade PA. Epigenetic remodelling upon FGFR inhibition. Nat Cell Biol. (2021) 23:1115–6. doi: 10.1038/s41556-021-00782-y 34737444

[29] Sanchez-Guixe M Hierro C Jimenez J Viaplana C Villacampa G Monelli E. High FGFR1-4 mRNA expression levels correlate with response to selective FGFR inhibitors in breast cancer. Clin Cancer Res. (2022) 28:137–49. doi: 10.1158/1078-0432.CCR-21-1810 34593528

[30] Formisano L Lu Y Servetto A Hanker AB Jansen VM Bauer JA. Aberrant FGFR signaling mediates resistance to CDK4/6 inhibitors in ER+ breast cancer. Nat Commun. (2019) 10:1373. doi: 10.1038/s41467-019-09068-2 30914635 PMC6435685

[31] Gautam P Jaiswal A Aittokallio T Al-Ali H Wennerberg K. Phenotypic screening combined with machine learning for efficient identification of breast cancer-selective therapeutic targets. Cell Chem Biol. (2019) 26:970–9. doi: 10.1016/j.chembiol.2019.03.011 PMC6642004 31056464

[32] Wei W Cao S Liu J Wang Y Song Q Leha A. Fibroblast growth factor receptor 4 as a prognostic indicator in triple-negative breast cancer. Transl Cancer Res. (2020) 9:6881–8. doi: 10.21037/tcr-20-1756 PMC8797274 35117296

[33] Krook MA Reeser JW Ernst G Barker H Wilberding M Li G. Fibroblast growth factor receptors in cancer: genetic alterations, diagnostics, therapeutic targets and mechanisms of resistance. Br J Cancer. (2021) 124:880–92. doi: 10.1038/s41416-020-01157-0 PMC7921129 33268819

[34] Nakamura IT Kohsaka S Ikegami M Ikeuchi H Ueno T Li K. Comprehensive functional evaluation of variants of fibroblast growth factor receptor genes in cancer. NPJ Precis Oncol. (2021) 5:66. doi: 10.1038/s41698-021-00204-0 34272467 PMC8285406

[35] Zingg D Bhin J Yemelyanenko J Kas SM Rolfs F Lutz C. Truncated FGFR2 is a clinically actionable oncogene in multiple cancers. Nature. (2022) 608:609–17. doi: 10.1038/s41586-022-05066-5 PMC9436779 35948633

[36] Gluz O Kolberg-Liedtke C Prat A Christgen M Gebauer D Kates R. Efficacy of deescalated chemotherapy according to PAM50 subtypes, immune and proliferation genes in triple-negative early breast cancer: Primary translational analysis of the WSG-ADAPT-TN trial. Int J Cancer. (2020) 146:262–71. doi: 10.1002/ijc.v146.1 31162838

[37] Frattini V Pagnotta SM Tala Fan JJ Russo MV Lee SB. A metabolic function of FGFR3-TACC3 gene fusions in cancer. Nature. (2018) 553:222–7. doi: 10.1038/nature25171 PMC5771419 29323298

[38] Chew NJ Nguyen EV Su SP Novy K Chan HC Nguyen LK. FGFR3 signaling and function in triple negative breast cancer. Cell Commun Signal. (2020) 18:13. doi: 10.1186/s12964-019-0486-4 31987043 PMC6986078

[39] Katoh M. Fibroblast growth factor receptors as treatment targets in clinical oncology. Nat Rev Clin Oncol. (2019) 16:105–22. doi: 10.1038/s41571-018-0115-y 30367139

[40] Lee HJ Seo AN Park SY Kim JY Park JY Yu JH. Low prognostic implication of fibroblast growth factor family activation in triple-negative breast cancer subsets. Ann Surg Oncol. (2014) 21:1561–8. doi: 10.1245/s10434-013-3456-x 24385208

[41] Bogatyrova O Mattsson J Ross EM Sanderson MP Backman M Botling J. FGFR1 overexpression in non-small cell lung cancer is mediated by genetic and epigenetic mechanisms and is a determinant of FGFR1 inhibitor response. Eur J Cancer. (2021) 151:136–49. doi: 10.1016/j.ejca.2021.04.005 33984662

[42] Li Y Qiu X Wang X Liu H Geck RC Tewari AK. FGFR-inhibitor-mediated dismissal of SWI/SNF complexes from YAP-dependent enhancers induces adaptive therapeutic resistance. Nat Cell Biol. (2021) 23:1187–98. doi: 10.1038/s41556-021-00781-z 34737445

[43] Suh J Kim DH Lee YH Jang J-H Surh Y-J. Fibroblast growth factor-2, derived from cancer-associated fibroblasts, stimulates growth and progression of human breast cancer cells via FGFR1 signaling. Mol Carcinog. (2020) 59:1028–40. doi: 10.1002/mc.23233 32557854

[44] Cazet AS Hui MN Elsworth BL Wu SZ Roden D Chan C-L. Targeting stromal remodeling and cancer stem cell plasticity overcomes chemoresistance in triple negative breast cancer. Nat Commun. (2018) 9:2897. doi: 10.1038/s41467-018-05220-6 30042390 PMC6057940

[45] Liu G Chen T Ding Z Wang Y Wei Y Wei X. Inhibition of FGF-FGFR and VEGF-VEGFR signalling in cancer treatment. Cell Prolif. (2021) 54:e13009. doi: 10.1111/cpr.13009 33655556 PMC8016646

[46] Adachi Y Kamiyama H Ichikawa K Fukushima S Ozawa Y Yamaguchi S. Inhibition of FGFR reactivates IFNgamma signaling in tumor cells to enhance the combined antitumor activity of lenvatinib with anti-PD-1 antibodies. Cancer Res. (2022) 82:292–306. doi: 10.1158/0008-5472.CAN-20-2426 34753772 PMC9397636

[47] Ichikawa K Watanabe MS Minoshima Y Matsui J Funahashi Y. Activated FGF2 signaling pathway in tumor vasculature is essential for acquired resistance to anti-VEGF therapy. Sci Rep. (2020) 10:2939. doi: 10.1038/s41598-020-59853-z 32076044 PMC7031295

[48] Pitt JM Marabelle A Eggermont A Soria J-C Kroemer G Zitvogel L. Targeting the tumor microenvironment: removing obstruction to anticancer immune responses and immunotherapy. Ann Oncol. (2016) 27:1482–92. doi: 10.1093/annonc/mdw168 27069014

[49] Majidpoor J Mortezaee K. The efficacy of PD-1/PD-L1 blockade in cold cancers and future perspectives. Clin Immunol. (2021) 226:108707. doi: 10.1016/j.clim.2021.108707 33662590

[50] Deng H Kan A Lyu N Mu L Han Y Liu L. Dual vascular endothelial growth factor receptor and fibroblast growth factor receptor inhibition elicits antitumor immunity and enhances programmed cell death-1 checkpoint blockade in hepatocellular carcinoma. Liver Cancer. (2020) 9:338–57. doi: 10.1159/000505695 PMC7325120 32647635

[51] Im JH Buzzelli JN Jones K Franchini F Gordon-Weeks A Markelc B. FGF2 alters macrophage polarization, tumour immunity and growth and can be targeted during radiotherapy. Nat Commun. (2020) 11:4064. doi: 10.1038/s41467-020-17914-x 32792542 PMC7426415

[52] Reed JR Stone MD Beadnell TC Ryu Y Griffin TJ Schwertfeger KL. Fibroblast growth factor receptor 1 activation in mammary tumor cells promotes macrophage recruitment in a CX3CL1-dependent manner. PloS One. (2012) 7:e45877. doi: 10.1371/journal.pone.0045877 23029290 PMC3454319

[53] Park MH Lee JS Yoon JH. High expression of CX3CL1 by tumor cells correlates with a good prognosis and increased tumor-infiltrating CD8+ T cells, natural killer cells, and dendritic cells in breast carcinoma. J Surg Oncol. (2012) 106:386–92. doi: 10.1002/jso.v106.4 22422195

[54] Holdman XB Welte T Rajapakshe K Pond A Coarfa C Mo Q. Upregulation of EGFR signaling is correlated with tumor stroma remodeling and tumor recurrence in FGFR1-driven breast cancer. Breast Cancer Res. (2015) 17:141. doi: 10.1186/s13058-015-0649-1 26581390 PMC4652386

[55] Akhand SS Liu Z Purdy SC Abdullah A Lin H Cresswell GM. Pharmacologic inhibition of FGFR modulates the metastatic immune microenvironment and promotes response to immune checkpoint blockade. Cancer Immunol Res. (2020) 8:1542–53. doi: 10.1158/2326-6066.CIR-20-0235 PMC7710538 33093218

[56] Welte T Kim IS Tian L Gao X Wang H Li J. Oncogenic mTOR signalling recruits myeloid-derived suppressor cells to promote tumour initiation. Nat Cell Biol. (2016) 18:632–44. doi: 10.1038/ncb3355 PMC4884142 27183469

[57] Denkert C von Minckwitz G Brase JC Sinn BV Gade S Kronenwett R. Tumor-infiltrating lymphocytes and response to neoadjuvant chemotherapy with or without carboplatin in human epidermal growth factor receptor 2-positive and triple-negative primary breast cancers. J Clin Oncol. (2015) 33:983–91. doi: 10.1200/JCO.2014.58.1967 25534375

[58] Weaver A Bossaer JB. Fibroblast growth factor receptor (FGFR) inhibitors: A review of a novel therapeutic class. J Oncol Pharm Pract. (2021) 27:702–10. doi: 10.1177/1078155220983425 33375902

[59] Dai S Zhou Z Chen Z Xu G Chen Y. Fibroblast growth factor receptors (FGFRs): structures and small molecule inhibitors. Cells. (2019) 8(6):614. doi: 10.3390/cells8060614 31216761 PMC6627960

[60] Sharpe R Pearson A Herrera-Abreu MT Johnson D Mackay A Welti JC. FGFR signaling promotes the growth of triple-negative and basal-like breast cancer cell lines both in *vitro* and in *vivo* . Clin Cancer Res. (2011) 17:5275–86. doi: 10.1158/1078-0432.CCR-10-2727 PMC3432447 21712446

[61] Ye T Wei X Yin T Xia Y Li D Shao B. Inhibition of FGFR signaling by PD173074 improves antitumor immunity and impairs breast cancer metastasis. Breast Cancer Res Treat. (2014) 143:435–46. doi: 10.1007/s10549-013-2829-y 24398778

[62] Itoh N Ornitz DM. Evolution of the Fgf and Fgfr gene families. Trends Genet. (2004) 20:563–9. doi: 10.1016/j.tig.2004.08.007 15475116

[63] Sootome H Fujita H Ito K Ochiiwa H Fujioka Y Ito K. Futibatinib is a novel irreversible FGFR 1-4 inhibitor that shows selective antitumor activity against FGFR-deregulated tumors. Cancer Res. (2020) 80:4986–97. doi: 10.1158/0008-5472.CAN-19-2568 32973082

[64] Meric-Bernstam F Bahleda R Hierro C Sanson M Bridgewater J Arkenau H-T. Futibatinib, an irreversible FGFR1-4 inhibitor, in patients with advanced solid tumors harboring FGF/FGFR aberrations: A phase I dose-expansion study. Cancer Discovery. (2022) 12:402–15. doi: 10.1158/2159-8290.CD-21-0697 PMC9762334 34551969

[65] Boichuk S Dunaev P Mustafin I Mani S Syuzov K Valeeva E. Infigratinib (BGJ 398), a pan-FGFR inhibitor, targets P-glycoprotein and increases chemotherapeutic-induced mortality of multidrug-resistant tumor cells. Biomedicines. (2022) 10(3):601. doi: 10.3390/biomedicines10030601 35327403 PMC8945560

[66] Tsimafeyeu I Ludes-Meyers J Stepanova E Daeyaert F Khochenkov D Joose J-P. Targeting FGFR2 with alofanib (RPT835) shows potent activity in tumour models. Eur J Cancer. (2016) 61:20–8. doi: 10.1016/j.ejca.2016.03.068 27136102

[67] Peng P Qiang X Li G Li L Ni S Yu Q. Tinengotinib (TT-00420), a novel spectrum-selective small-molecule kinase inhibitor, is highly active against triple-negative breast cancer. Mol Cancer Ther. (2023) 22:205–14. doi: 10.1158/1535-7163.MCT-22-0012 PMC9890131 36223547

[68] Piha-Paul SA Xu B Dumbrava EE Fu S Karp DD Meric-Bernstam F. First-in-human phase I study of tinengotinib (TT-00420), a multiple kinase inhibitor, as a single agent in patients with advanced solid tumors. Oncologist. (2024) 29:e514–25. doi: 10.1093/oncolo/oyad338 PMC10994248 38297981

[69] Liao M Zhou J Wride K Lepley D Cameron T Sale M. Population pharmacokinetic modeling of lucitanib in patients with advanced cancer. Eur J Drug Metab Pharmacokinet. (2022) 47:711–23. doi: 10.1007/s13318-022-00773-w PMC9399017 35844029

[70] Bello E Taraboletti G Colella G Zucchetti M Forestieri D Licandro SA. The tyrosine kinase inhibitor E-3810 combined with paclitaxel inhibits the growth of advanced-stage triple-negative breast cancer xenografts. Mol Cancer Ther. (2013) 12:131–40. doi: 10.1158/1535-7163.MCT-12-0275-T 23270924

[71] Bai A Meetze K Vo NY Kollipara S Mazsa EK Winston WM. GP369, an FGFR2-IIIb-specific antibody, exhibits potent antitumor activity against human cancers driven by activated FGFR2 signaling. Cancer Res. (2010) 70:7630–9. doi: 10.1158/0008-5472.CAN-10-1489 20709759

[72] Catenacci D Rasco D Lee J Rha SY Lee K-W Bang YJ. Phase I escalation and expansion study of bemarituzumab (FPA144) in patients with advanced solid tumors and FGFR2b-selected gastroesophageal adenocarcinoma. J Clin Oncol. (2020) 38:2418–26. doi: 10.1200/JCO.19.01834 PMC7367551 32167861

[73] van Brummelen E Levchenko E Domine M Fennell DA Kindler HL Viteri S. A phase Ib study of GSK3052230, an FGF ligand trap in combination with pemetrexed and cisplatin in patients with Malignant pleural mesothelioma. Invest New Drugs. (2020) 38:457–67. doi: 10.1007/s10637-019-00783-7 PMC6898757 31065954

[74] Oki E Makiyama A Miyamoto Y Kotaka M Kawanaka H Miwa K. Trifluridine/tipiracil plus bevacizumab as a first-line treatment for elderly patients with metastatic colorectal cancer (KSCC1602): A multicenter phase II trial. Cancer Med. (2021) 10:454–61. doi: 10.1002/cam4.v10.2 PMC7877360 33249761

[75] Palakurthi S Kuraguchi M Zacharek SJ Zudaire E Huang W Bonal DM. The combined effect of FGFR inhibition and PD-1 blockade promotes tumor-intrinsic induction of antitumor immunity. Cancer Immunol Res. (2019) 7:1457–71. doi: 10.1158/2326-6066.CIR-18-0595 31331945

[76] Ruan R Li L Li X Huang C Zhang Z Zhong H. Unleashing the potential of combining FGFR inhibitor and immune checkpoint blockade for FGF/FGFR signaling in tumor microenvironment. Mol Cancer. (2023) 22:60. doi: 10.1186/s12943-023-01761-7 36966334 PMC10039534

[77] Ou X Tan Y Xie J Yuan J Deng X Shao R. Methylation of GPRC5A promotes liver metastasis and docetaxel resistance through activating mTOR signaling pathway in triple negative breast cancer. Drug Resist Update. (2024) 73:101063. doi: 10.1016/j.drup.2024.101063 38335844

[78] Bhola NE Jansen VM Koch JP Li H Formisano L Williams JA. Treatment of triple-negative breast cancer with TORC1/2 inhibitors sustains a drug-resistant and notch-dependent cancer stem cell population. Cancer Res. (2016) 76:440–52. doi: 10.1158/0008-5472.CAN-15-1640-T PMC4715956 26676751

